# Cross-Domain Text Mining of Pathophysiological Processes Associated with Diabetic Kidney Disease

**DOI:** 10.3390/ijms25084503

**Published:** 2024-04-19

**Authors:** Krutika Patidar, Jennifer H. Deng, Cassie S. Mitchell, Ashlee N. Ford Versypt

**Affiliations:** 1 Department of Chemical and Biological Engineering, University at Buffalo, Buffalo, NY 14260, USA; 2 Wallace H. Coulter Department of Biomedical Engineering, Georgia Institute of Technology and Emory University, Atlanta, GA 30332, USA; 3 Center for Machine Learning at Georgia Tech, Georgia Institute of Technology, Atlanta, GA 30332, USA; 4 Department of Biomedical Engineering, University at Buffalo, Buffalo, NY 14260, USA; 5 Institute for Artificial Intelligence and Data Science, University at Buffalo, Buffalo, NY 14260, USA

**Keywords:** diabetic kidney disease, immune response, glomerular endothelial cells, biomedical text mining, disease relatedness, functional ontology, machine learning, artificial intelligence

## Abstract

Diabetic kidney disease (DKD) is the leading cause of end-stage renal disease worldwide. This study’s goal was to identify the signaling drivers and pathways that modulate glomerular endothelial dysfunction in DKD via artificial intelligence-enabled literature-based discovery. Cross-domain text mining of 33+ million PubMed articles was performed with SemNet 2.0 to identify and rank multi-scalar and multi-factorial pathophysiological concepts related to DKD. A set of identified relevant genes and proteins that regulate different pathological events associated with DKD were analyzed and ranked using normalized mean HeteSim scores. High-ranking genes and proteins intersected three domains—DKD, the immune response, and glomerular endothelial cells. The top 10% of ranked concepts were mapped to the following biological functions: angiogenesis, apoptotic processes, cell adhesion, chemotaxis, growth factor signaling, vascular permeability, the nitric oxide response, oxidative stress, the cytokine response, macrophage signaling, NFκB factor activity, the TLR pathway, glucose metabolism, the inflammatory response, the ERK/MAPK signaling response, the JAK/STAT pathway, the T-cell-mediated response, the WNT/β-catenin pathway, the renin–angiotensin system, and NADPH oxidase activity. High-ranking genes and proteins were used to generate a protein–protein interaction network. The study results prioritized interactions or molecules involved in dysregulated signaling in DKD, which can be further assessed through biochemical network models or experiments.

## 1. Introduction

Diabetic kidney disease (DKD) is a major microvascular complication in the kidney that affects patients with type I diabetes and type II diabetes. Approximately 20–50% of patients with type II diabetes will develop DKD [1]. DKD can lead to a decline in kidney function and has the potential to develop into chronic kidney disease or end-stage renal disease (ESRD) [2,3]. A high clinical and socio-economic impact of DKD is burdensome because of the risk of progression to ESRD and other related comorbidities [4]. The progression of chronic and end-stage renal failure is estimated to affect over 10% of the general population, or more than 800 million people, worldwide [5]. The pathophysiology of DKD is multi-factorial and characterized by metabolic impairment, an uncontrolled inflammatory response, increased apoptosis, and tissue fibrosis [4,6]. In diabetes, aberrant glucose metabolism leads to dysregulation of the immune response and signaling [6,7,8]. Metabolic abnormalities activate the mononuclear phagocyte system, which releases pro-inflammatory cytokines and paracrine signals, leading to immune cell infiltration [6,7,8,9]. In the early stages of diabetes, glomerular and tubular cells have increased expression of tumor necrosis factor (TNF) α, interleukin (IL)-6, IL-1, and adhesion molecules [10,11]. Previous experimental studies showed that hyperglycemia promotes the imbalance in macrophage phenotypes and chronic glucose exacerbates the regulation of inflammatory responses [12,13]. The dysregulated signaling response leads to progressive kidney damage through loss of glomerular endothelial fenestrations, thickening of the basement membrane, the detachment of podocyte foot processes, mesangial matrix expansion, and glomerular fibrosis [7,14,15,16,17]. For excellent illustrations of the pathological processes, readers are referred to [7,8,16,17].

Several mathematical models have demonstrated the pathophysiological processes affected at the cellular or tissue scale due to underlying kidney disease [9,15,18,19,20] (Table A1). Mathematical models integrate literature-based information into a quantitative framework, identifying knowledge gaps and generating new experimentally testable hypotheses. Studies have demonstrated that it is relevant to comprehend previously applied knowledge and relate it across disease domains to identify potential mechanisms and targeted treatment. A recent mathematical model of the disease etiology of glomerular fibrosis in DKD has successfully adapted and related the key attributes of glomerular fibrosis in lupus nephritis, a type of kidney disease [15]. Hyperglycemia-induced podocyte injury in DKD has been previously modeled using the local renin–angiotensin system in renal podocyte cells [18]. It is well known that dysregulation of the renin–angiotensin–aldosterone system is a phenomenon implicated in the pathophysiology of both renal disorders [6] and cardiovascular disorders, such as hypertension and congestive heart failure [21].

Mathematical models have also studied the pathophysiological interplay of the kidney with other organ disorders related to cardiavascular conditions [22], cancer [23], or bone metabolism [24,25] at a multi-scale and multi-organ level using existing individual cardiac, renal, or metabolic bone disorder models (Table A1). Examples of such models vary across a spectrum of first-principles-based mathematical models to complex network models. In each case, the parameters are typically estimated from the literature, and decisions must be made as to what concepts should be included. A few examples of such relevant models of kidney disease are highlighted below.

A relevant mathematical model was developed to examine the progression from tubulointerstitial inflammation to fibrosis in lupus nephritis using limited available knowledge of the pathogenesis of inflammation in lupus nephritis [20]. The model was built using the first principles of engineering and physics with parameters estimated using experimental literature. The advantage of such a first-principles model is the inherent interpretability. However, many pathophysiological concepts were simplified to make the mathematics tractable.

A different approach to mathematically modeling kidney disease used a signaling network topology and regulatory motifs in podocytes. The model contributed to the understanding of the establishment and maintenance of the morphologically distinct state of the podocytes in the kidney [19]. An extensive literature survey of cell culture studies and proteomics data was performed to build network models with varying levels of detail and provide insights into treatment strategies for kidney disease [19].

**Figure 1:**
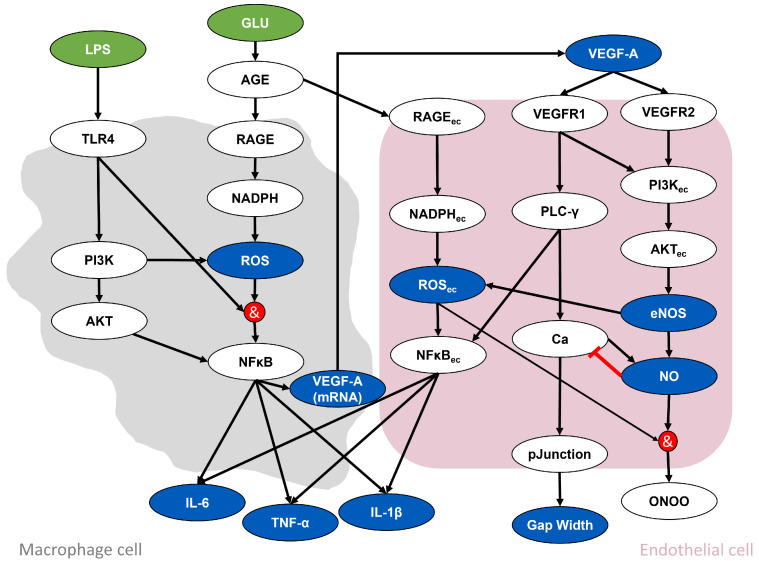
A multi-cellular protein–protein interaction network of crosstalk between macrophages (**left**, grey shape) and glomerular endothelial cells (**right**, pink shape) stimulated with glucose (GLU) and lipopolysaccharide (LPS), a pro-inflammatory stimulus, was created in our previous work through manual curation of the literature [9]. Green nodes (ovals) are input nodes, blue nodes are output nodes, and white nodes are regulatory nodes. Black arrows are activating interactions, a red line with a flat-head arrow is an inhibiting interaction, and red circles indicate logic AND gates. An OR logic rule connects two or more edges to a subsequent node throughout the network unless indicated otherwise by an AND logic gate. The subscript (ec) denotes an intracellular species expressed in glomerular endothelial cells. IL-6, TNF-α, IL-1β, and VEGF-A are protein levels expressed in extracellular space. ROS, ROSec, VEGF-A (mRNA), and NO are expressed within the cells. The Gap Width node denotes a fractional change in the glomerular endothelial cell fenestration size. The pJunction node represents the phosphorylated junction protein levels. TLR: toll-like receptor. AGE: advanced glycation end product. RAGE: receptor of advanced glycation end product. NADPH: nicotinamide adenine dinucleotide phosphate. NFκB: nuclear factor kappa B. IL: interleukin. TNF: tumor necrosis factor. PI3K: phosphoinositide 3-kinase. AKT: serine/threonine-specific protein kinases. ROS: reactive oxygen species. VEGF: vascular endothelial growth factor. VEGFR: vascular endothelial growth factor receptor. PLC: phospholipase C. NO: nitric oxide. ONOO: peroxynitrite. eNOS: endothelial nitric oxide synthase. Ca: calcium. Reprinted/adapted with permission from Ref. [9], 2023, K. Patidar and A. N. Ford Versypt.

Another example of an approach to mathematically modeling kidney disease used a protein–protein interaction network model (Figure 1). The model was proposed using interaction databases and produced in vitro experimental data to understand the progression of early signs of endothelial dysfunction in DKD [9]. The multi-cell network was a manually curated, simplified network of pathway interactions and signaling molecules that affect glomerular endothelial fenestrations in the diabetic kidney. However, that biochemical network [9] only incorporated a subset of the relevant pathways, interactions, or molecules governing DKD progression that were derived from relevant publications.

A further way to understand DKD-related pathways is the use of bioinformatics analyses across different cell types in DKD. A single-cell renal endothelial transcriptomic atlas using differential gene expression revealed the alteration of signaling pathways including oxidative phosphorylation and growth factor signaling in renal endothelial cell subtypes [26]. The recent application of single-cell RNA and bulk RNA sequencing data to study the co-stimulatory interactions between renal endothelial cells and macrophages has been useful in identifying the immunological markers of DKD [27,28].

In short, regardless of the mathematical method implemented, the relevant pathway interconnections to include are typically decided using domain knowledge and knowledge acquired from manual literature curation. However, a human cannot read all possible sources of important relationships that should be included in a multi-scalar, multi-factorial model of a complex pathophysiology like DKD. Moreover, signaling molecules in the disease pathway may be located further away from the target molecule or event of interest within the scientific literature [29]. Thus, a manual literature search may be insufficient for finding relationships not only within the DKD literature, but also in the cross-domain literature (cardiology, endocrinology, immunology, etc.) where relevant concepts and relationships may reside. In contrast, advanced high-throughput artificial intelligence approaches to literature-based discovery (LBD) can provide a holistic understanding of relevant dysregulated pathways and molecules. LBD approaches enabled by text mining infer disparate sources of information [30] at a scale not otherwise possible.

An example of a recent, comprehensive LBD approach to identifying and ranking relevant concepts is SemNet 2.0 [31,32]. SemNet is an open-source framework consisting of a knowledge graph that identifies and ranks the most important concepts to a user-defined target concept(s) (e.g., keyword). The graph consists of relationships extracted from 33+ million published articles in the PubMed database. The nodes are concepts defined by the Unified Medical Language System. The framework uses SemMedDB as a relationship extraction system for making the graph [33]. The unsupervised learning ranking algorithm within SemNet 2.0 examines relationship patterns in the literature to rank cross-domain concepts with respect to the user-defined concept(s) [31]. SemNet 2.0 has been used for drug repurposing for COVID-19 [34] and Parkinson’s disease [35], identifying unknown disease mechanisms of resistant hypertension following COVID-19 infection [36], predicting adverse events from chronic tyrosine kinase inhibitor therapy in chronic myeloid leukemia [29], and identifying clinical features by which to better stratify chemotherapy-related infection risk in pediatric acute leukemia [37].

**Figure 2:**
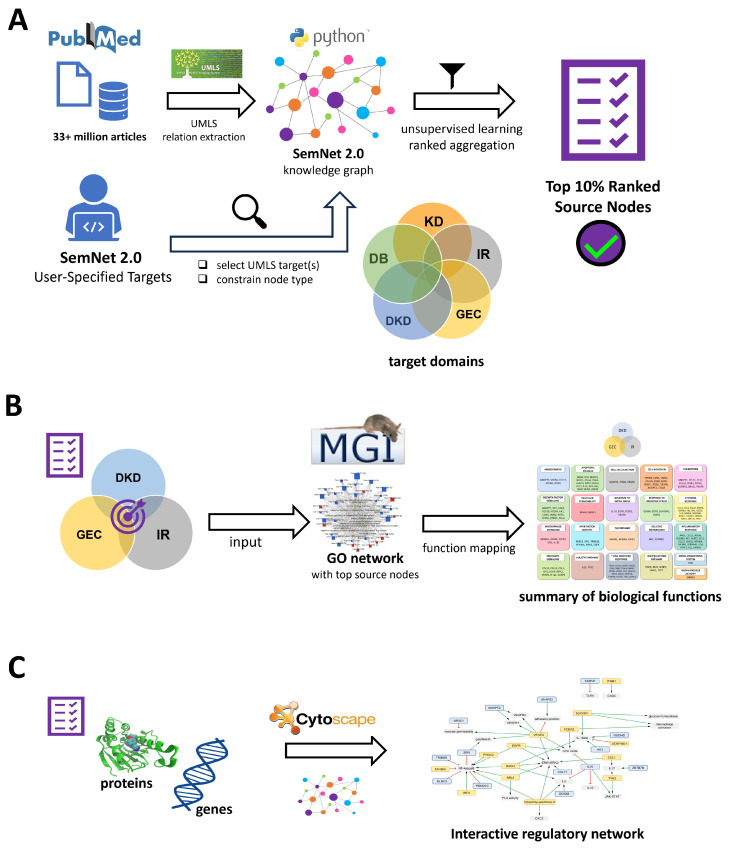
The baseline data used in this project included a knowledge graph with semantic text relationships extracted from 33+ million PubMed articles. The workflow consisted of (**A**) performing a SemNet 2.0 analysis to identify the top-ranked source nodes across domains, (**B**) performing a Gene Ontology (GO) network analysis to summarize the biological functions of the top-ranked source nodes, and (**C**) visualizing regulatory relationships from the top-ranked genes and proteins using Cytoscape [38,39]. Note that the target domains are labeled as diabetes (DB), kidney disease (KD), immune response (IR), diabetic kidney disease (DKD), and glomerular endothelial cells (GEC).

The objective of the present study was to identify and rank cross-domain concepts that comprise the complex multi-factorial and multi-scalar nature of DKD using advanced artificial intelligence-assisted LBD software. In this study, SemNet 2.0 was used to identify and rank critical signaling molecules associated with the glucose- and inflammation-mediated development and progression of DKD. The general workflow is shown in Figure 2. This comprehensive evaluation enabled the prioritization of highly ranked concepts, as well as the identification of concepts or phenomena missing from current mathematical network models of DKD. Our study results indicate that the utilized LBD approach provided a less biased and more comprehensive manner of integrating cross-domain knowledge into the mechanistic understanding of DKD.

The contributions of this work are as follows:

- The developed workflow with SemNet 2.0 and additional post hoc analysis with Gene Ontology (GO) networks and Cytoscape support the integrative prioritization of literature relationships that advance the study of DKD.
- The top-ranked source nodes identified by SemNet 2.0 enable the more comprehensive construction of protein–protein interactions, the efficient modeling of biochemical pathways, and the expedited testing of literature-based hypotheses through experiments.
- The application of artificial intelligence-enabled LBD techniques to DKD research enables faster integration and processing of new biomedical literature towards secondary analyses that improve drug discovery and target treatment design.

## 2. Results

The LBD results from SemNet 2.0 identified and ranked the shared concepts among five Unified Medical Language System (UMLS)-defined pathophysiological concepts relevant to the study of DKD, including DKD itself, as well as diabetes (DB), kidney disease (KD), the immune response (IR), and glomerular endothelial cells (GEC). The intersecting top 10% of source genes and proteins at the intersection of the DKD, IR, and GEC domains were used to map the biological functions with GO network analysis. Finally, the mapped biological functions and potential regulatory relationships of the identified source nodes were visualized in a Cytoscape [38,39] network to expedite subsequent biochemical models. Collectively, these results provide a comprehensive, prioritized summary of key relationships important to the study and treatment of DKD. The specific results are discussed below with deeper supporting context.

### 2.1. Source Nodes Revealed by Cross-Domain Analysis

**Figure 3:**
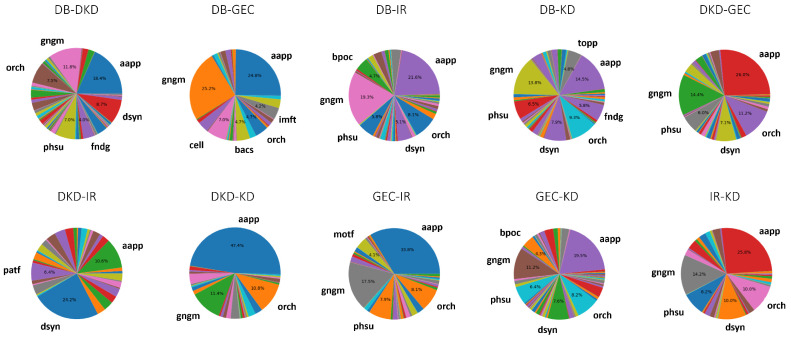
Semantic type distribution of source nodes identified at the intersection of each pairwise domain. Arbitrary colors are assigned to each pie chart segment. Labels are provided in the diagram for types that reach at least 4% share of a distribution. gngm: gene or genome. aapp: amino acid, peptide, or protein. dsyn: disease or syndrome. phsu: pharmacological substance. fndg: finding. imft: immunologic factor. orch: organic chemical. cell: cell. bacs: biologically active substance. patf: pathologic function. bpoc: body part, organ, or organ component. topp: therapeutic or preventive procedures. DB: diabetes domain. DKD: diabetic kidney disease domain. GEC: glomerular endothelial cells domain. IR: immune response domain. KD: kidney disease domain.

**Figure 4:**
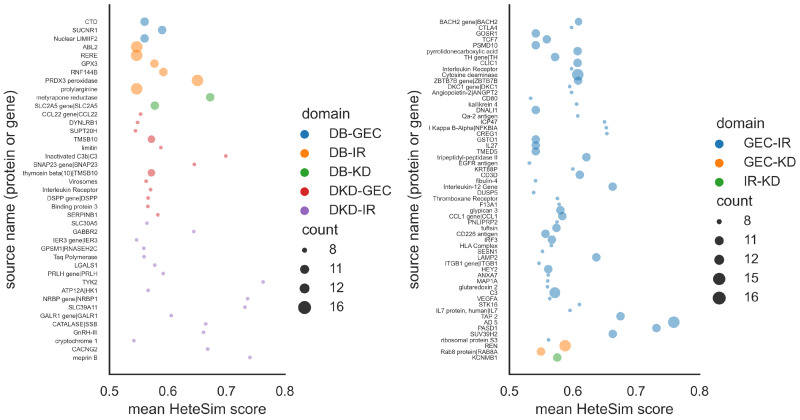
Bubble plot of source nodes identified by SemNet 2.0 at the intersection of each pairwise domain. Source nodes (genes or proteins) are shown on the vertical axis, each pairwise domain is denoted by a different bubble color, and the frequency (count) of each source node is distinguished by the bubble size. Source nodes are either genes or proteins. For clarity, the source nodes are presented in two plots (**left** and **right**). DB: diabetes domain. DKD: diabetic kidney disease domain. GEC: glomerular endothelial cells domain. IR: immune response domain. KD: kidney disease domain. Definitions of the source node abbreviations are provided in the Supplementary File S2.

The distribution of source nodes in each semantic type for each pairwise domain analysis is shown in Figure 3. For instance, the distribution of source nodes at the intersection of both the DB and DKD domains was found in the cross-domain analysis between the DB and DKD (DB-DKD) domains. Higher distributions of intersecting nodes of the semantic type gene (gngm) or type protein (aapp) were observed in each pairwise domain (Figure 3). Nodes belonging to the gngm or aapp semantic type from the top 10% of source nodes were selected for further evaluation of their biological themes and functional relevance in the progression of DKD. The distributions of these top 10% of source nodes in each paired domain are shown and characterized by their mean HeteSim scores and counts (Figure 4). The source nodes are represented by a unique bubble with a color indicative of the pairwise domain and a size indicative of the frequency of node occurrence (Figure 4). The top 10% of source nodes obtained from the post hoc analysis belonged mainly to the GEC, DKD, and IR domains. As seen in Figure 4, more source nodes were present at the intersection of the GEC-IR, DKD-IR, and DKD-GEC domains relative to the other pairwise domains. Most of the identified source nodes with a high mean HeteSim score were present at the intersection of the DKD-IR and GEC-IR domains (Figure 4).

### 2.2. Biological Themes Associated with Top 10% of Source Nodes

**Figure 5:**
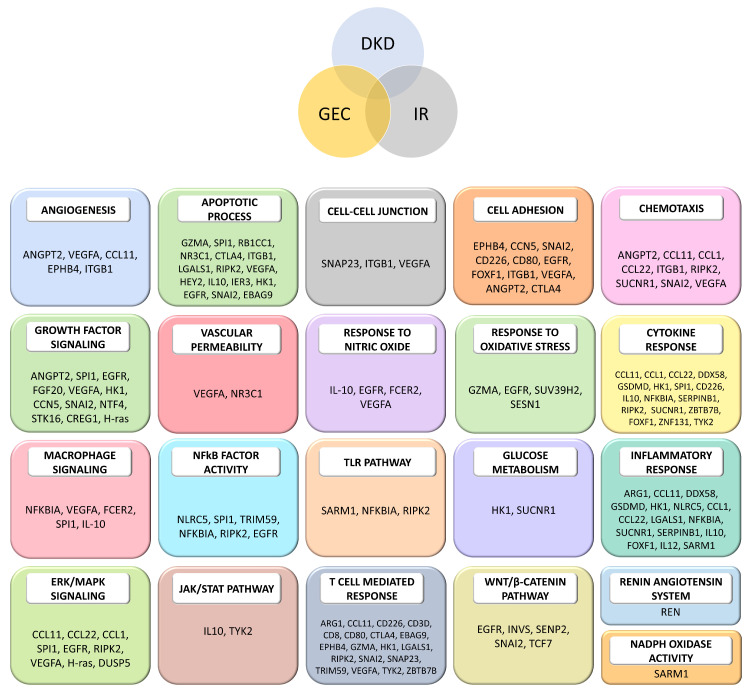
The top 10% of genes and proteins that intersect in the three domains—diabetic kidney disease (DKD), immune response (IR), and glomerular endothelial cells (GEC)—are grouped into their biological processes (block headers). NFkB: nuclear factor κ B. TLR: toll-like receptor. ERK: extracellular signal-regulated kinase. MAPK: mitogen-activated protein kinase. JAK: Janus kinase. STAT: signal transducer and activator of transcription. WNT: wingless/integrated. NADPH: nicotinamide adenine dinucleotide phosphate. The colors for the domains correspond to those used in Figure 9. The colors for the biological processes are arbitrary and are purely for aesthetic purposes. Definitions of the source node (gene and protein) abbreviations are provided in the Supplementary File S2.

A unique functional ontology or biological process was mapped to each of the top 10% of source genes and proteins using the mouse genome informatics (MGI) term mapper [40,41]. A total of 99 genes and 117 proteins comprised the intersection of the DKD, IR, and GEC domains, which were mapped to 32 unique ontology IDs that described their biological functions. Figure 5 represents the identified genes and proteins and their respective mapped unique ontology IDs. These 21 unique ontologies (Figure 5) are also among the most common biological themes associated with DKD progression, a dysregulated immune response and inflammation, and the functional and structural integrity of endothelial cells. About 25% of the intersecting source nodes were found to be related to apoptotic function, 25% of the source nodes were related to the T-cell-mediated response, 22% of the intersecting source nodes were related to the cytokine response and inflammatory response, and 15% of the source nodes were related to growth factor signaling. Moreover, cell adhesion, chemotaxis, and the ERK/MAPK signaling pathway were also among the well-represented functional ontologies.

**Figure 6:**
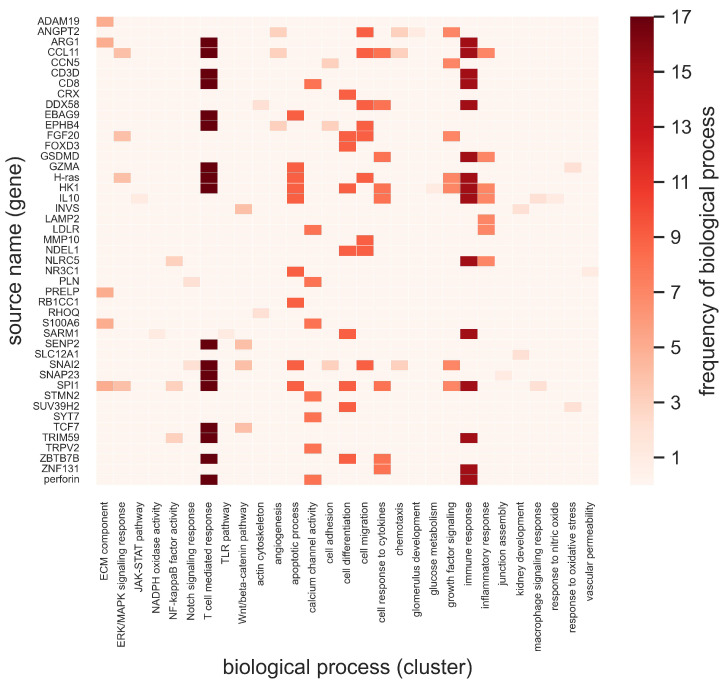
Source node (gene) names and their biological processes (term label) in the diabetic kidney disease (DKD), immune response (IR), and glomerular endothelial cells (GEC) domains. The frequency (term count) of each biological process (*x*-axis) is color-coded in the range of 0–17. ECM: extracellular matrix. ERK: extracellular signal-regulated kinase. MAPK: mitogen-activated protein kinase. JAK: Janus kinase. STAT: signal transducer and activator of transcription. NADPH: nicotinamide adenine dinucleotide phosphate. NF: nuclear factor. TLR: toll-like receptor. Wnt: wingless/integrated. Definitions of the source node abbreviations are provided in the Supplementary File S2.

**Figure 7:**
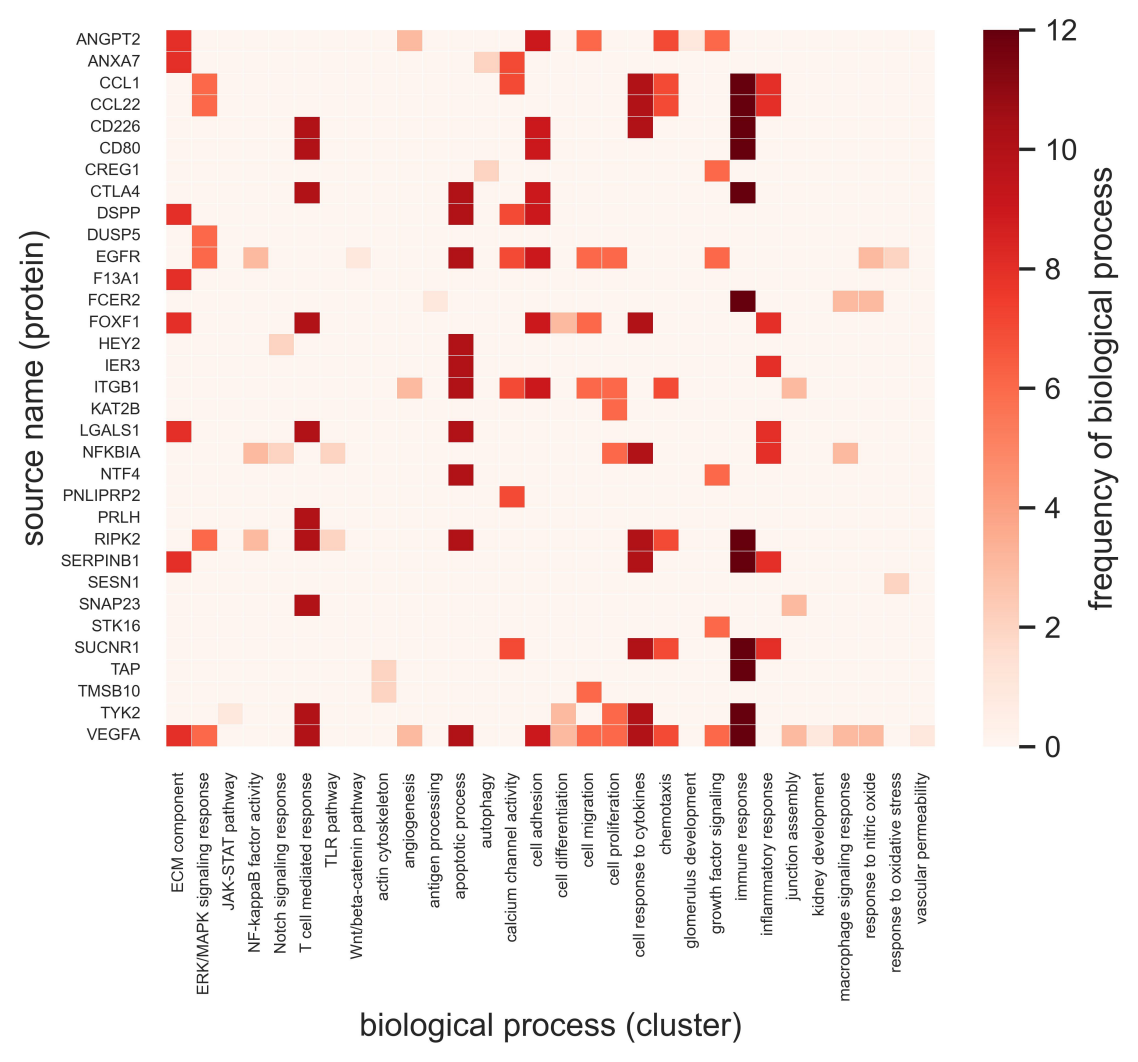
Source node (protein) names and their biological processes (term label) in the diabetic kidney disease (DKD), immune response (IR), and glomerular endothelial cells (GEC) domains. The frequency (term count) of each process (*x*-axis) is color-coded in the range 0–12. ECM: extracellular matrix. ERK: extracellular signal-regulated kinase. MAPK: mitogen-activated protein kinase. JAK: Janus kinase. STAT: signal transducer and activator of transcription. NADPH: nicotinamide adenine dinucleotide phosphate. NF: nuclear factor. TLR: toll-like receptor. Wnt: wingless/integrated. Definitions of the source node abbreviations are provided in the Supplementary File S2.

Further, the biological functions associated with these identified source genes (Figure 6) and source proteins (Figure 7) are summarized. The top 10% of source nodes were associated with biological processes, including the ERK/MAPK signaling cascade, the JAK/STAT pathway, NF-κB factor activity, growth factor signaling, the Wnt/β-catenin pathway, and the TLR pathway. Moreover, these genes and proteins were found to have multiple functional roles. The frequency of a biological process or function associated with a source node qualitatively describes the relative importance of the biological function. Figure 6 and Figure 7 show heatmaps of the top 10% of identified source nodes and their respective biological processes specified as a unique ontology ID. The frequencies of these processes are represented by color bars on the right. These source nodes in the GEC, IR, and DKD domains play a crucial role in the immune response, the T-cell-mediated response, the cytokine response, apoptosis, and cell adhesion, among other critical biological functions (Figure 6 and Figure 7). The biological processes, including the TLR pathway, the immune response, apoptotic processes, calcium channel activity, and the response to cytokines, were relatively more prevalent processes associated with the identified source genes (Figure 6). The biological processes of the T-cell-mediated response, apoptotic processes, cell adhesion, the response to cytokines, chemotaxis, and growth factor signaling were relatively more prevalent processes associated with the identified source proteins (Figure 7).

**Figure A1:**
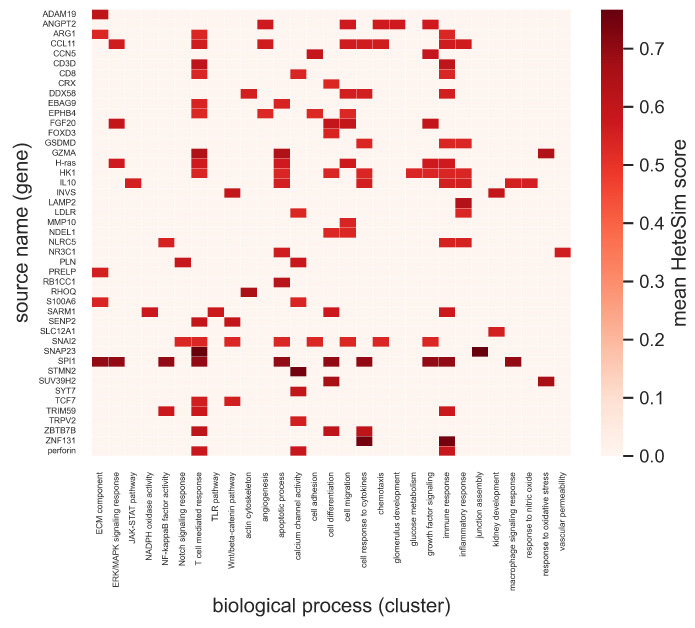
Source node (gene) names and their biological processes (term label) in the diabetic kidney disease (DKD), immune response (IR), and glomerular endothelial cells (GEC) domains. The mean HeteSim score of each gene from the SemNet 2.0 simulation is represented by the color bar on the right. ECM: extracellular matrix. ERK: extracellular signal-regulated kinase. MAPK: mitogen-activated protein kinase. JAK: Janus kinase. STAT: signal transducer and activator of transcription. NADPH: nicotinamide adenine dinucleotide phosphate. NF: nuclear factor. TLR: toll-like receptor. Wnt: wingless/integrated. Definitions of the source node abbreviations are provided in the Supplementary File S2.

**Figure A2:**
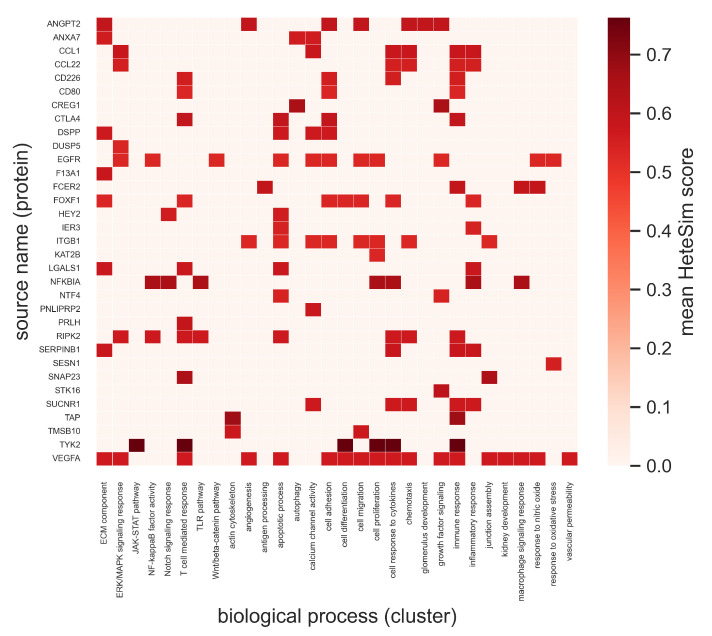
Source nodes (protein) names and their biological processes (term label) in the diabetic kidney disease (DKD), immune response (IR), and glomerular endothelial cells (GEC) domains. The mean HeteSim score of each protein from the SemNet 2.0 simulation is represented by the color bar on the right. ECM: extracellular matrix. ERK: extracellular signal-regulated kinase. MAPK: mitogen-activated protein kinase. JAK: Janus kinase. STAT: signal transducer and activator of transcription. NADPH: nicotinamide adenine dinucleotide phosphate. NF: nuclear factor. TLR: toll-like receptor. Wnt: wingless/integrated. Definitions of the source node abbreviations are provided in the Supplementary File S2.

**Figure A3:**
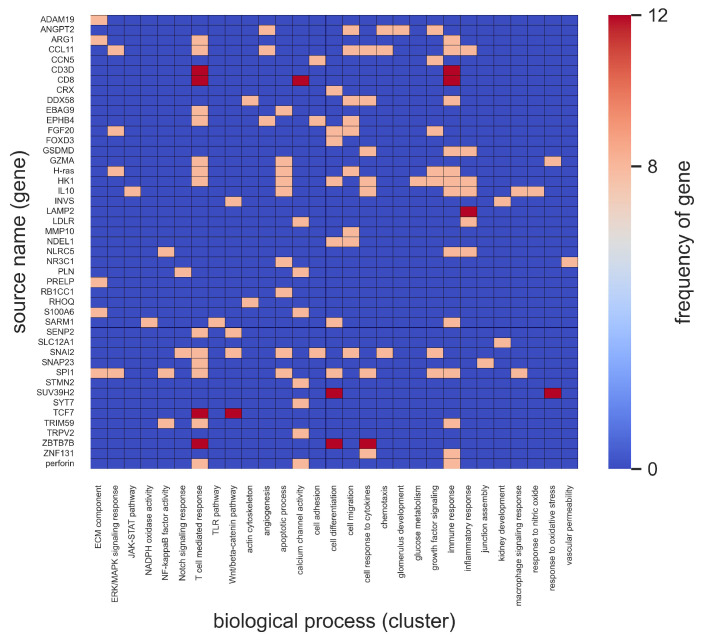
Source node (gene) names and their biological processes (term label) in the diabetic kidney disease (DKD), immune response (IR), and glomerular endothelial cells (GEC) domains. The frequency (count) of each source gene (*y*-axis) is color-coded. ECM: extracellular matrix. ERK: extracellular signal-regulated kinase. MAPK: mitogen-activated protein kinase. JAK: Janus kinase. STAT: signal transducer and activator of transcription. NADPH: nicotinamide adenine dinucleotide phosphate. NF: nuclear factor. TLR: toll-like receptor. Wnt: wingless/integrated. Definitions of the source node abbreviations are provided in the Supplementary File S2.

**Figure A4:**
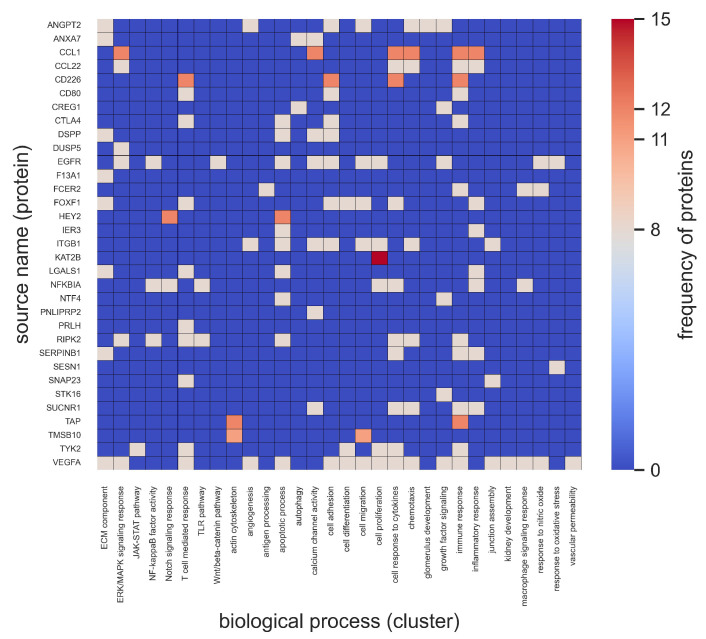
Source node (protein) names and their biological processes (term label) in the diabetic kidney disease (DKD), immune response (IR), and glomerular endothelial cells (GEC) domains. The frequency (count) of each source protein (*y*-axis) is color-coded. ECM: extracellular matrix. ERK: extracellular signal-regulated kinase. MAPK: mitogen-activated protein kinase. JAK: Janus kinase. STAT: signal transducer and activator of transcription. NADPH: nicotinamide adenine dinucleotide phosphate. NF: nuclear factor. TLR: toll-like receptor. Wnt: wingless/integrated. Definitions of the source node abbreviations are provided in the Supplementary File S2.

The mean HeteSim scores of the identified genes (Figure A1) and proteins were provided and (Figure A2) mapped to their unique ontology IDs. The SPI1, SNAP23, STMN2, and ZNF131 genes and TYK2, NFKBIA, and CREG1 proteins had the highest mean HeteSim scores and were closely related to the user-specified targets. The CD3D, CD8, LAMP2, SUV39H2, TCF7, and ZBTB7B genes (Figure A3) frequently recurred in the cross-domain analysis and were associated with the T-cell-mediated response, the Wnt/β-catenin pathway, cell differentiation, the immune response, the response to cytokines, and the response to oxidative stress. The CCL1, CD226, HEY2, TAP2, and TMSB10 proteins (Figure A4) frequently occurred at the intersection of the DKD, GEC, and IR domains and were associated with biological processes like the JAK/STAT pathway, the Notch signaling response, the T-cell-mediated response, cell migration, the response to cytokines, chemotaxis, and the immune response.

### 2.3. Interaction Network in Cytoscape

**Figure 8:**
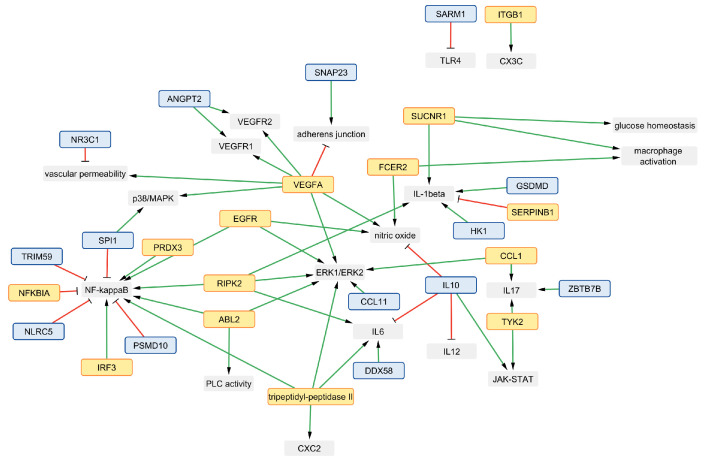
Regulatory relationship between top 10% of identified source nodes and signaling molecules generated in Cytoscape [38,39]. The grey blocks are signaling molecules/outcomes. The yellow blocks with an orange border are source proteins, and the blue blocks with a dark blue border are genes obtained from SemNet 2.0 analysis. A green arrow indicates a positive regulatory relationship between two nodes, and a red line with a flat end indicates a negative regulatory relationship. Signaling molecules (grey blocks) are defined as follows: TLR: toll-like receptor. NF-kappaB: nuclear factor-κB. ERK: extracellular signal-regulated kinase. MAPK: mitogen-activated protein kinase. JAK: Janus kinase. STAT: signal transducer and activator of transcription. VEGF: vascular endothelial growth factor. VEGFR: vascular endothelial growth factor receptor. CX3C: CX3C-chemokine. CX2C: CX2C-chemokine. IL: interleukin. PLC: phospholipase. Additional definitions for other sources are provided in the Supplementary File S2.

The interaction network between source genes or proteins and their respective regulatory relationships, generated in Cytoscape [38,39], is shown in Figure 8. The interaction network assembles the annotated outputs from the semantic network together. Figure 8 shows a positive regulatory relationship and negative regulatory relationship, through green arrows and red lines with a flat end, respectively, between source proteins and genes. The source genes and proteins are shown with a blue node and a yellow node, respectively. Upon analyzing the interaction network, it was observed that NF-κB, a transcription factor, is involved in numerous signaling events, including the inflammatory response; it is positively regulated by the PRDX3, EGFR, RIPK2, ABL2, and IRF3 genes and negatively regulated by the SPI1, TRIM59, NLRC5, and PSMD10 proteins and the NFKBIA gene. The VEGFA gene is central to the regulation of VEGF receptor 1 (VEGFR1), VEGF receptor 2 (VEGFR2), the adherens junction, nitric oxide, p38/MAPK signaling kinase, and ERK1/ERK2 signaling kinase. Vascular endothelial development and growth in endothelial cells heavily rely on nitric oxide [9,17,42], which is positively influenced by the VEGFA, EGFR, and FCER2 genes and negatively influenced by the the IL-10 protein. RIPK2 and DDX58, as well as GSDMD, RIPK2, and HK1, play positive roles in regulating pro-inflammatory cytokines like IL-6 and IL-1β. The anti-inflammatory IL-10 gene was correlated with the response to inflammatory cytokines, IL-1β, IL-6, IL-17, and IL-12. SUCNR1 is involved in both glucose homeostasis and macrophage activation and is a potential link in understanding glucose-mediated macrophage cell polarization [43]. The adherens junction proteins are responsible for regulating the endothelial cell–cell junction and vascular permeability in healthy and diseased states [9,44,45,46,47]. Our analysis identified that adherens junction proteins are positively regulated by the SNAP23 protein and negatively regulated by the VEGFA gene. The NR3C1 protein encodes a glucocorticoid receptor and was found to play a role in reducing vascular permeability within endothelial cells. The FCER2 gene was one of the identified genes involved in positive regulation of macrophage activation.

## 3. Discussion

The biomedical literature is a continuously growing repository of complex and deeply interconnected information. Despite powerful, user-friendly scientific databases, it is difficult for scientists and clinicians to extract useful information in their niche from these large and complex databases [34]. SemNet 2.0, an open-source literature-based discovery technique applied in this study, assists scientists and clinicians by leveraging the power of biomedical text mining to guide their research and development efforts. In this study, novel cross-domain text mining with SemNet 2.0 identified signaling molecules and pathways that are often studied in relation to diabetes, the immune response, kidney disease, and dysfunction of glomerular endothelial cells. The cross-domain analyses determined the relatedness between five pathological events by identifying significant source nodes that are mutually shared by these pathological events. The pairwise cross-domain analyses also determined the distribution of these source nodes across different semantic types (Figure 3).

### 3.1. Top-Ranked Intersecting GEC-IR-DKD Nodes

Among the top 10% of predicted source nodes, 77 source nodes were common among the GEC, IR, and DKD domains (Figure 4). The source genes or proteins with relatively high mean HeteSim scores were highly associated with and prevalent in the GEC, DKD, and IR domains (Figure 4). The observed intersection (Figure 5) indicates the importance of studying the synergistic interaction between the immune system and glomerular endothelial cells to better understand the early stage of DKD progression.

#### 3.1.1. Top-Ranked Intersecting Cellular Functions and Signaling Processes

The top 10% of intersecting source nodes were associated with various cellular functions and cellular signaling responses. The cellular functions include angiogenesis, glucose metabolism, cell apoptosis, cell–cell junction integrity, and cell adhesion. The cellular signaling responses include growth factor signaling, the response to nitric oxide, the response to oxidative stress, the cytokine response, macrophage signaling, the TLR pathway, the T-cell-mediated response, NFκB factor activity, ERK/MAPK signaling, the JAK/STAT pathway, the WNT/β-catenin pathway, and NADPH oxidase activity. Several of these pathways are known to be implicated in GEC injury, inflammation, and fibrosis associated with DKD [6,8,11,17]. Similarly, the analyses suggested that the apoptotic processes, the response to cytokines, the T-cell-mediated response, the immune response, calcium channel activity, and growth factor signaling were the most frequent and active biological processes in DKD (Figure 6 and Figure 7).

- Role of T-cells. This finding is consistent with other studies that report T-cells as being the most studied immune cells that infiltrate kidney tissues and trigger inflammatory responses in DKD [48,49,50]. Given the prevalent role of T-cells and the T-cell-mediated cytokine response in DKD [28,51,52], their highly ranked importance by the unsupervised ranking algorithm in SemNet 2.0 was expected.
- Role of calcium. Increased calcium channel activity exerts significant vascular and tubular effects on the kidneys, which leads to the enhancement of glomerular filtration rate (GFR) and renal blood flow (RBF) [53,54,55].
- Role of VEGF. Growth factor signaling via VEGF, fibroblast growth factors, transforming growth factor-β, and insulin-like growth factors in diabetes and diabetic kidney disease has been studied in detail [56,57]. VEGF is a potent angiogenic and vascular permeability factor and is responsible for endothelial cell proliferation and differentiation and increased permeability [9,58]. VEGF also maintains endothelial cell homeostasis, and a disturbance in basal VEGF levels is implicated in diabetes-related complications, including kidney disease [59]. Specifically, VEGF-A is associated with macrophage or monocyte differentiation, which suggests its role in the macrophage response in pathological conditions [9,42,57,58].
- Role of TGF. An increase or decrease in the production of transforming growth factor -β1 (TGF-β1) has been associated with diabetic nephropathy and retinopathy [56]. The expression of TGF-β1 is increased in endothelial cells, which, in turn, triggers the activation of TGF receptors, namely TGFBR2 and TGFBR3, on B lymphocytes, podocytes, glomerular endothelial cells, and mesangial cells, leading to epithelial–mesenchymal transition and fibrosis in the development of diabetic nephropathy [17,57,59]. Insulin-like growth factor-I is a naturally occurring single-chain polypeptide that has been widely used in the treatment of diabetic glomerular and renal tubular injuries [56,57,60].

#### 3.1.2. Top-Ranked Intersecting Genes

The identified genes were involved in multiple functional roles. Highlights for some of the top-ranked genes are discussed below in the context of the literature.

- Role of succinate receptor 1 (SUCNR1). SUCNR1 is involved in both glucose homeostasis and macrophage activation. SUCNR1 is an extracellular receptor activated by succinate, and SUCNR1 accumulation in macrophages is known to activate the pro-inflammatory response [43]. Moreover, the role of SUCNR1 has been suggested in the development of fibrosis in diabetes mellitus and other diabetes-related complications such as diabetic retinopathy and metabolic syndrome [61]. SUCNR1 can serve as a potential link in understanding glucose-mediated macrophage cell polarization.
- Role of hexokinase 1 (HK1). The HK1 gene encodes a ubiquitous form of hexokinase, which localizes to the outside membrane of mitochondria. Mutations in HK1 have been associated with hemolytic anemia due to hexokinase deficiency. However, its role in DKD is more likely linked to dysregulated glucose metabolism. HK1 is also associated with the cytokine response, the inflammatory response, and growth factor signaling [62].
- Role of ephrin. Ephrin receptors make up the largest subgroup of the receptor tyrosine kinase family, which have a key role in vascular regulation. SemNet 2.0 has previously highlighted the role of tyrosine kinase pathways in resistant hypertension [36]. The protein encoded by this gene binds to ephrin-B2 and plays an essential role in vascular development. Ephrin receptor EPHB4 is associated with angiogenesis in DKD, the immune response, and GEC. EPHB4 receptor interactions between endothelial cells and monocytes/macrophages are relevant for vascular development [57,59]. The inhibition of proteins in the ephrin B family prevents endothelial cell sprouting and initiates disorders in endothelial cell assembly [59].
- Role of serpin family B member 1 (SERPINB1). SERPINB1 is among the serpin protein families that are found in GEC. SERPINB1 acts primarily to protect the cells from proteases released into the cytoplasm during stress [63]. The results presented suggest SERPINB1’s involvement in the inflammatory cytokine response. Previous research has suggested serpin proteins to be associated with macrophage motility as well [59].
- Role of integrins. Integrins regulate many biological processes, such as cell growth, migration, and signaling and cytokine activation, thereby contributing to inflammation and angiogenesis [64]. In the present study, integrin ITGB1 was associated with several cellular functions: angiogenesis, apoptosis, cell–cell junction integrity, and cell adhesion (Figure 5). Studies have also suggested combined treatment strategies through the inhibition of both ITGB and ITGA integrins to reduce macrophage filtration into the glomeruli [59,65]. Such compelling evidence suggests that ITGB1 may have the potential to be a clinical marker for the prognosis of glomerular diseases, immune cell infiltration, and glomerular endothelial viability [59].

#### 3.1.3. Top-Ranked Intersecting Proteins

Among the top 10% of predicted source proteins, the TYK2, CREG1, NFKBIA, and SNAP23 proteins were highly associated with the user-specific target nodes based on the calculated mean HeteSim scores (Figure A2).

- Role of tyrosine kinsase 2 (TYK2). Previous studies found an association of TYK2 candidate with type 1 diabetes mellitus and a role of TYK2 in regulating apoptotic and pro-inflammatory pathways in pancreatic β-cells through modulation of the type I interferon signaling pathway [66,67]. Likewise, tyrosine kinase inhibitor drugs were previously predicted by SemNet 2.0 to be associated with hyperglycemia in patients who were not initially diabetic [29].
- Role of cellular repressor of E1A stimulated genes 1 (CREG1). CREG1 has been studied rigorously in relation to glucose uptake, renal dysfunction, angiogenesis, and diabetes-related comorbidity [68,69,70].
- Role of NFKBIA. NFKBIA regulates the activity of NFκB, which plays a role in processes such as the accumulation of advanced glycation end products and activation of the renin–angiotensin system pathways, protein kinase C, and oxidative stress in diabetic nephropathy [71].
- Role of synaptosome-associated protein 23 (SNAP23). Our analyses also identified SNAP23 associated with adherens junction assembly in correlation to GEC, the immune response, and DKD [72]. SPI1 and SNAP23 were genes highly associated with the immune response, cell differentiation, cell migration, the response to cytokines, and apoptotic processes (Figure 6 and Figure A1). Some studies have previously identified SPI1 gene involvement in regulatory mechanisms in DKD, but this may need more experimental verification [73]. The SNAP23 gene is relatively abundant in the kidney and primarily involved in exocytosis [74]. SNAP23 has been shown to reduce proteinuria, reduce podocyte foot process fusion, and reduce endothelial cell damage upon the inhibition of SNAP23-mediated exocytosis [75].

### 3.2. Visualization of Literature-Based Discovery Network Predictions

Here the SemNet 2.0 analysis was conducted, and the prediction was visualized as an interaction network using Cytoscape [38,39]. Such a representation of source nodes using Cytoscape allowed for a straightforward interpretation of regulatory relationships in our generated data set. The comprehensive text mining analysis provided potential candidates involved in dysregulated signaling events (Figure 8) that can be used to address the limitations of our existing network model [9]. Relevant similarities were identified between the SemNet 2.0 findings and the previous network (Figure 1). Both networks share some common pathophysiological outcomes and signaling nodes involving TLR4, VEGF-A, VEGFR1, VEGFR2, IL-1β, IL-6, NO, PLC, NF-κB, the adherens junction, vascular permeability, and macrophage activation (Figure 1 and Figure 8). SemNet 2.0 analysis is useful for recognizing proteins or genes that regulate these signaling nodes and/or pathophysiological events of interest. The predicted source genes and proteins can be further studied through network-based computational approaches and mechanistic modeling. The regulatory relationship visualized in Cytoscape could be a useful starting point to build a network-based model or identify interconnections between genes or proteins that may enable us to overcome the knowledge gaps or limitations of existing or published network models. There are various open-source and paid alternatives to Cytoscape, including Gephi [76], Tableau [77], NodeXL [78], and Neo4j [79], which can be used to visualize data obtained from text mining techniques. CompositeView has many similarities to Cytoscape and has been successfully implemented and customized to examine SemNet and SemNet 2.0 results [80]. A detailed comparison of the strengths and limitations of CompositeView against other similar software has been provided previously [80].

### 3.3. Comparing LBD Networks to Traditional Bioinformatics Networks

Including comprehensive biomedical literature in the cross-domain analysis provided breadth in the mechanistic understanding of disease progression, which is often not achievable through manual literature searches. This study demonstrated the compatibility and ease of use of the LBD tool SemNet 2.0, with various pieces of open-source bioinformatics software, to efficiently gather and assemble information that can be useful in the field of systems biology. Specifically, the present study outlined a process and case study by which to compare the similarities and knowledge gaps between signaling motifs obtained from SemNet 2.0 and previously published traditional signaling networks.

Another modality that this study’s overall analysis can be qualitatively compared to is the pathway enrichment analysis of differentially expressed genes. Recent meta-analyses of gene expression datasets for diabetic nephropathy obtained the following top terms from their pathway enrichment analyses: immune system, extracellular matrix organization, hemostasis, signal transduction, and platelet activation by Hojjati et al. [81] and immune activation, T-cell activation, and cell adhesion by Zhong et al. [28]. While these are not exactly the terms our analysis yielded, they are related. For example, hemostasis broadly encompasses several of the cellular functions we listed in Section 3.1.1. Likewise, signal transduction lumps together many of the cellular signaling responses we itemized in Section 3.1.1. Immune system includes effects that resulted from our analysis, such as the response to cytokines, the T-cell-mediated response, and the immune response. Extracellular matrix organization is highly related to TGF and fibrosis. Among the previously identified diagnostic markers for DKD from Zhong et al. [28], tenascin C (TNC), tissue inhibitor metalloproteinase 1 (TIMP1), and tropomyosin 1 (TPM1) were also identified by the cross-domain analyses here (refer to raw data “combined.csv” in [82]). All 15 of the hub differentially expressed genes listed in Hojjati et al. [81] were also identified here (see raw data “combined.csv” in [82]).

### 3.4. Limitations and Future Directions

Biomedical text mining and similarity-based clustering analyses have their limitations. The clustering of these biomedical concepts or nodes based on similarity represents similarity in the patterns of associations with the user-specified target node. Thus, the similarity-based association of source to target depends on the amount and quality of literature data [83]. The implementation of additional link prediction algorithms with SemNet 2.0, as was performed by McCoy and colleagues to use SemNet 2.0 to predict COVID-19 drugs while the virus was new and was the subject of minimal studies [34], is one way to overcome this limitation. Regardless, a larger sample size of data reduces any bias from any lesser-quality publications. On the other hand, the user can control the loss of information when less evidence for a subject is available in the literature [31,83].

SemNet 2.0 is a methodology for ranking relevance and relatedness among nodes in a knowledge graph. This is similar to link prediction models, which enable the inference of novel relationships from existing edges and nodes in a knowledge graph. Performing link prediction leads to more nuanced search queries that build on SemNet simulation results. The Python library Pykeen has built-in functionality that can predict the head, relation, or tail (h, r, t) for an incomplete triple [84]. For example, given the incomplete triple (?, r, t), candidates for the head are scored and ranked based on KG embedding models such as RotatE, TransE, and ComplEx. Link prediction can be used for future research with the SemNet simulation results presented here.

Moreover, with advancements in single-cell transcriptomics, near-single-cell proteomics, and spatial metabolomics, there is emerging evidence and data for kidney tissues and infiltrating macrophages [57,85,86,87]. The widespread availability of such integrated and high-quality datasets [85] will enable better information gain when using LBD techniques. Future work could involve validating or comparing the findings from SemNet 2.0 to single-cell RNA sequencing data [57,85,86,87]. Integrating data within and across domains remains a big challenge due to heterogeneity. Recent and ongoing progress towards the collection, standardization, and integration of various metadata variables from data resources, including Kidney Tissue Atlas Ontology, Precision Medicine Metadata Ontology, and the Human Reference Atlas, has proven effective in identifying kidney-specific gene biomarkers and cell types [88].

Currently, a wide knowledge gap exists between biology and drug development, which results in sub-optimal treatment options against DKD [89]. Although glycemic control treatments are useful in the management of DKD to some extent, there is still potential for the discovery of new treatment strategies targeting inflammation, oxidative stress, fibrosis, and other pathological events [15,90]. Future applications may involve applying cross-domain analysis for the identification of pathological mechanisms, treatment strategies, and plausible hypotheses for DKD treatment and management. The proposed LBD technique can aid in bridging the knowledge gaps between DKD etiology and treatment. Applying LBD techniques to DKD research will enable faster processing of novel and actionable knowledge from vast, diverse, and seemingly disconnected fragments of information and the utilization of processed information towards treatment design [91]. The Kidney Precision Medicine Project (KPMP) has extensively contributed to the representation of kidney phenotype terms for acute and chronic kidney disease and increased the ability to improve personalized treatment. Future analysis of the KPMP data using LBD may be feasible in extracting information by profiling and integrating clinical, pathological, cellular, and molecular characteristics associated with the increasing pool of patients with specific diseases [88]. The highly associated genes or proteins observed at the intersection of the DKD, IR, and GEC domains could be used in future assessment through either experimental validation or a mathematical model.

## 4. Materials and Methods

The present study used advanced artificial intelligence-based text mining techniques to identify relevant signaling molecules and their relation to glucose-mediated inflammation in DKD. The general workflow is shown in Figure 2. First, SemNet 2.0 simulations were performed to identify the top-ranked nodes across multiple relevant domains using a knowledge graph of semantic relationships extracted from 33+ PubMed articles (Figure 2A). The top-ranked nodes represent the most relevant concepts to DKD and its related pathophysiology. Next, using the top-ranked nodes, a GO network analysis was performed to obtain a functional map of the biological processes (Figure 2B). This functional map provides an intuitive means to summarize the thousands of relevant nodes into a format that enables mechanistic hypothesis formulation and testing. Finally, a regulatory interaction network was visualized in Cytoscape [38,39] using the top-ranked nodes and their corresponding semantic relationships from the SemNet knowledge graph (Figure 2C). In short, the regulatory interaction network provides the granular information necessary for the construction of subsequent biochemical models or protein interaction networks.

**Figure 9:**
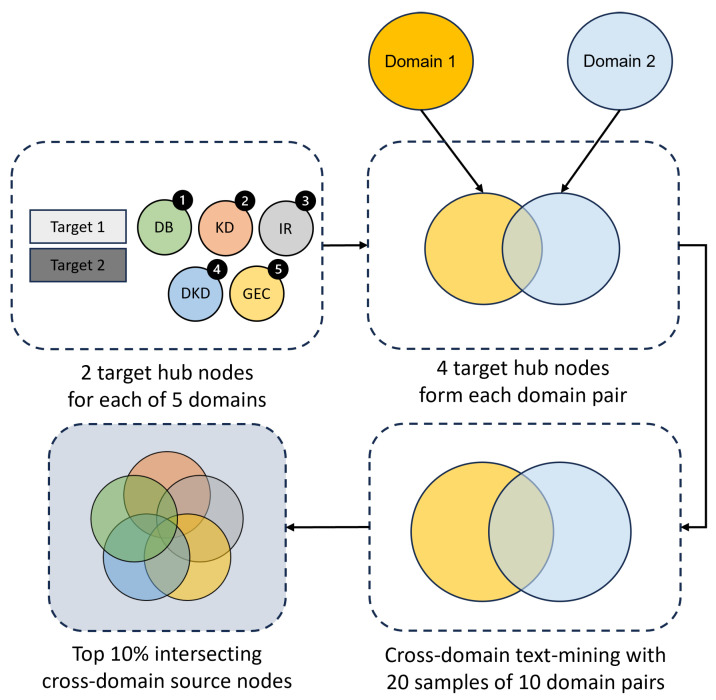
Workflow of cross-domain analyses in SemNet 2.0 performed to identify intersecting source nodes across five domains: diabetes (DB), kidney disease (KD), immune response (IR), diabetic kidney disease (DKD), and glomerular endothelial cells (GEC). Colored circles represent different domains, which are also numbered here. Illustrated in the first box, the first step was to randomly select two target nodes from each of the five domains. The specifics of the first step is illustrated in the next box, where each domain pair formed a combination of four target hub nodes. This information was provided as input to SemNet 2.0 to perform runs on each of these domain pairs. The process of generating four domain pairs was repeated 50 times, such that each of the ten unique domain pairs was sampled 20 times for a total of 200 SemNet simulations. This cross-domain text-mining process is represented by the third box. The final box illustrates that the SemNet simulations yielded intersecting cross-domain source nodes, and the top 10% of these source nodes were analyzed further. See Section 4.2 for details in the text.

Specifically, SemNet 2.0 was used to perform a cross-domain analysis across the following five disease domains: diabetes (DB), kidney disease (KD), immune response (IR), glomerular endothelial cells (GEC), and DKD (Figure 9). The user-specified target nodes for each domain were chosen from observed interactions in the network model and published studies [9]. For instance, the binding of toll-like receptors (TLRs) is one of the key determinants of the immune response. Therefore, it was considered as one of the target nodes in this study. A complete list of target nodes is provided in the Supplementary File S1.

### 4.1. SemNet 2.0

SemNet 2.0 [31] is a piece of open-source software that ingests publicly available text relationships from PubMed and the National Library of Medicine [33] to perform LBD tasks. SemNet 2.0 uses a heterogeneous semantic network to provide consistent and valuable categorization of all concepts represented in the UMLS metathesaurus, and the UMLS provides a universal ontology to relate concepts from the biomedical literature [92]. More information on the semantic networks and their types can be found in the UMLS reference manual [92]. SemNet 2.0 queries a biomedical knowledge graph composed of semantic triples extracted from PubMed’s 33 million abstracts. The original SemNet was proposed in 2019 by Mitchell and Sedler [83] and was later optimized by Kirkpatrick and colleagues in 2022 [31]. Each semantic triple consists of a head, a relation, and a tail, where the head and tail entities are the nodes, and the relation is a directed edge. The underlying mathematical theory and computational implementation of SemNet 2.0 can be found in the original publication [31]. SemNet 2.0 is summarized below in the context of its use in the present study.

SemNet 2.0 is available as Python code and uses natural language processing to identify source nodes relevant to user-specified target nodes. The source nodes are the set of nodes that the target nodes share in common; that is, they are reachable within the search depth and metapath length, which are search parameters defined in the next section. Each node is a biomedical concept, as defined in the UMLS, with a type such as “disease or syndrome” (dsyn), “amino acid, peptide, or protein” (aapp), etc. There are 133 types and 54 relations. Each directed edge encodes a relation, such as treats, affects, inhibits, etc.

**Figure 10:**
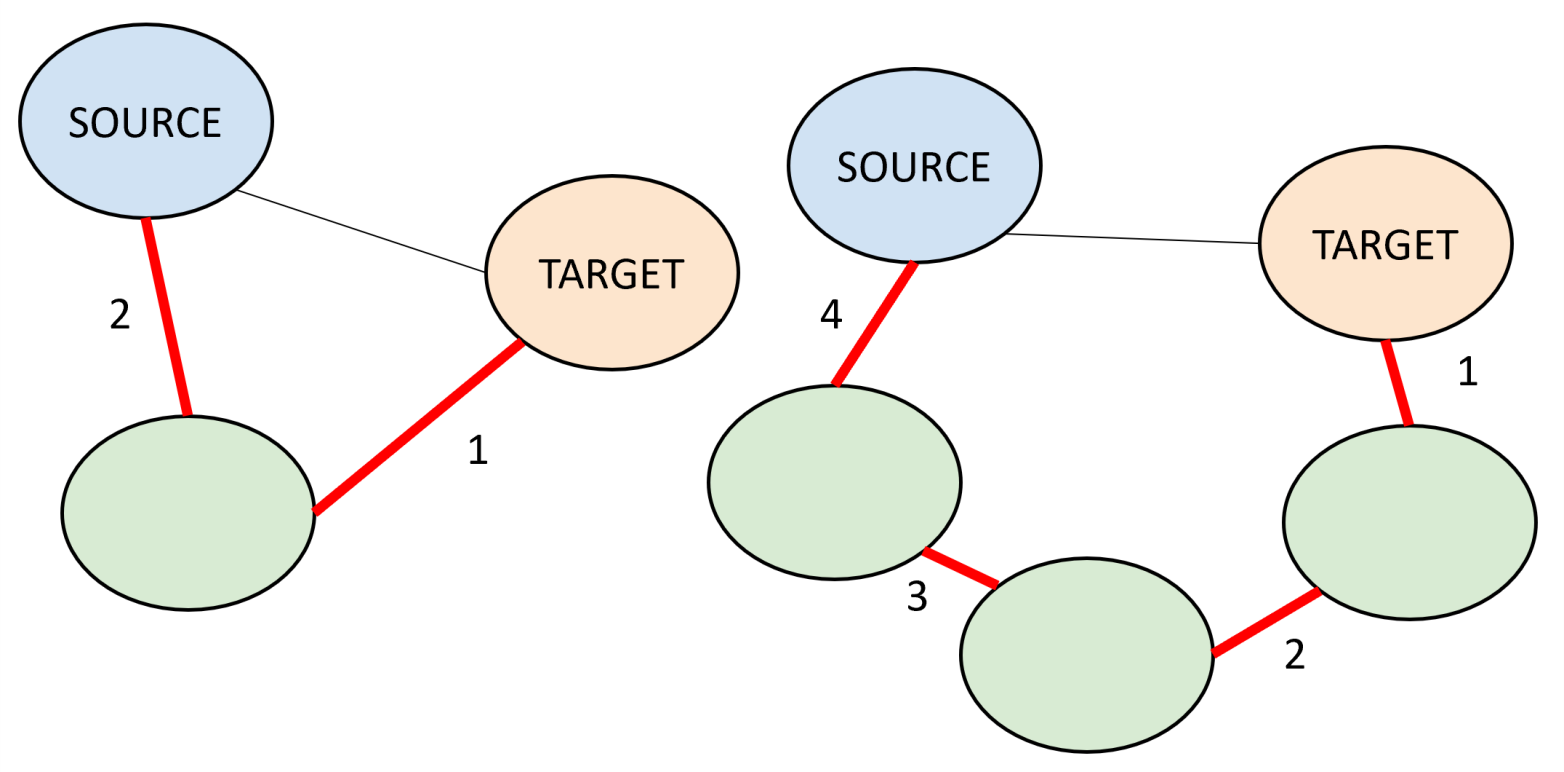
Two subgraphs with nodes (ovals) connected by edges (lines). The blue ovals are source nodes, the orange ovals are target nodes, and unlabeled green ovals represent nodes in the subgraph that are neither source nor target nodes. Red lines show the HeteSim metapath between target and source nodes, and the numbers near the lines provide running counts of the HeteSim metapath length that increases with each green node between the target and the source. In the subgraph on the left, the source node is at a depth of 1 away from the target, but the HeteSim metapath length is 2. On the right, the same source node is also at a depth of 1 away, but the HeteSim metapath length is 4.

The user defines four inputs: the target nodes, source node types, search depth, and metapath length. Target nodes are the nodes of interest, and SemNet 2.0 queries surrounding nodes that are connected to those nodes. The source node types can be restricted to certain semantic types, such as dsyn or aapp. Search depth is the number of hops away from the source node (Figure 10). For a given target node, a search depth of 1 finds all adjacent nodes directly connected to it. It is ideal to increase the search depth to find novel results, as the connections of neighbors to target nodes are more prominent and commonly acknowledged in the scientific literature. Metapath length is the total path from a target to a source node. Multiple paths can be consolidated into a single metapath based on the types of source nodes. Hypothetically, an infinite number of paths can be used to connect a target to a source node, and the metapath length can add a constraint for identifying relevant or innovative pathways. Increasing both metrics can drastically expand the scope of the search. As such, the metapath length and search depth inputs are actually key model parameters that can change the model results.

SemNet 2.0 calculates a metric called HeteSim to quantify the relevance between a source node and target node [31]. HeteSim was developed to quantify relevance in heterogeneous networks [93]. There are two ways to calculate HeteSim: deterministic and randomized. The deterministic HeteSim was used in all SemNet 2.0 simulations in the present study to enhance accuracy at the expense of computational speed. HeteSim can be further characterized by an exact (deterministic) mean or approximate mean. The exact mean is found by aggregating the HeteSim scores of multiple target nodes to the same source node. The approximate mean has a performance advantage over the exact mean, especially for metapaths of greater length. For the simulations here, the exact HeteSim mean was used.

HeteSim is calculated by determining the cosine similarity between two probability vectors. Let *x* be defined as the path length between a given target node and a source node. HeteSim takes the middle layer of nodes or the nodes at an x/2 path length away from the target. From the target and source nodes, weights of 1 are distributed evenly across nodes. Each subsequent layer continually redistributes the weights until the middle layer is reached. A left probability vector and a right probability vector are generated from either side. The cosine similarity is calculated between them, which is the HeteSim score. When combining the results from multiple SemNet 2.0 simulations, the mean HeteSim scores are normalized and the percentiles ranked to adjust for differences in node count, path count, etc. The normalization process allows the simulation results to be directly compared.

As described above, SemNet 2.0 uses unsupervised learning rank aggregation to calculate the importance of a source node using the HeteSim score. Because SemNet 2.0 is an unsupervised model, there is no ground truth set of rankings by which to explicitly compare them. Domain knowledge provides an important layer of validation. The results of SemNet 2.0 studies have been successfully evaluated in COVID-19 [34,36], Parkinson’s disease [35], chronic myeloid leukemia [29], and pediatric acute leukemia [37]. In fact, the majority of repurposed drug candidates recommended by SemNet early in the pandemic advanced to successful clinical trials as adjuvant therapies [94].

Note that the data for SemNet 2.0 simulations are based on processed semantic relationships from SemMedDB [31]. All data in the knowledge graph are available to be queried. However, the identified source nodes are limited by the search parameters, namely the search depth and metapath length parameters, as described above. The ranking results produced for a given query will remain consistent unless (1) a different (updated) version of the knowledge graph is deployed that contains additional new literature relationships or (2) the user changes the target node input(s) or specified search parameters.

SemNet 2.0 contains around 100 million semantic predictions (subject, object, predicate triples) extracted from PubMed articles. SemNet 2.0 separates the papers from their links and aggregates relation triples. SemMedDB is the basis of SemNet’s corpus of entities and relations [33].

Other biomedical-domain knowledge graphs exist, such as KnowLife, which uses UMLS as its dictionary and 13 binary relations [95]. It encompasses entities in health and life sciences, built from various web sources, including online communities. PubMed Knowledge Graph comprises entities from 29 million PubMed abstracts, in addition to providing granular-level details about the articles themselves, including each author’s educational background and affiliation history [96]. PrimeKG draws from only 20 resources, but it describes over 17,000 diseases and around 4 million relationships, with a focus on precision medicine analysis [97]. This knowledge graph allows the user to examine helpful indications and contraindications for drugs and how they impact disease progression. For drug repurposing, the model DREAMwalk—Drug Repurposing through Exploring Associations using Multi-layer random walk—uses “guilt-by-association” between drugs and diseases to generate hypothetical drug and disease node sequences, using a novel multi-layer approach that leverages node semantic neighbors [98]. Nonetheless, the unique properties of SemNet 2.0 made it the best choice for the present study.

### 4.2. SemNet 2.0 Simulations for DKD

Several relevant user-specified target nodes for each domain are given in the Supplementary File S1. Each of these targets was assumed to be a “hub node”, which is a singular node that is well connected in a graph. These were based on prior domain knowledge. Networks of hub nodes enable improved cross-domain analysis by functionally increasing the search depth in areas of the knowledge graph of chief interest [29,36].

In general, a SemNet 2.0 run is performed on cross-domain pairs to find the source nodes in common between four target nodes. To sample the user-specified target nodes between pairs of domains, the first step was to randomly select two target hub nodes from each of the five domains, resulting in each domain pair having a combination of four target hub nodes (Figure 9). Five domains with two hub nodes each yielded ten hub nodes and four domain pairs. A SemNet run was performed on each of these domain pairs. The process of generating four domain pairs was repeated 50 times, such that each of the ten unique domain pairs was sampled 20 times (Figure 9) for a total of 200 SemNet simulations. The search depth was 2, and the metapath length was 3. Due to computational limitations, if the number of source nodes exceeded 1000, a random sample of the source nodes was taken such that no more than 1000 nodes had their HeteSim scores calculated and ranked. If the number of source nodes exceeded 1500 or was less than 10, the random combination of target nodes was deemed unproductive, and a new simulation with a different set of target nodes was performed.

Some simulations had several hundreds of nodes within the <1500 range, but they were limited by a certain target node (e.g., “Disorder of mineral metabolism”). Throughout this study, each node or concept’s name and semantic type follow the UMLS ontology. SemNet 2.0 predicted source nodes associated with the target nodes and ranked these source nodes based on the optimized HeteSim similarity metric [31]. The association of the source nodes with user-specified target nodes in each pairwise domain was measured using a mean HeteSim score, calculated by averaging the HeteSim score of recurring nodes.

An exhaustive list of predicted source nodes was obtained at the intersection of each pairwise domain analysis. These identified source nodes were categorized into semantic types such as genes, proteins, and enzymes. These source nodes appeared more than once in each pairwise domain analysis. Each unique source node’s occurrence was counted as the source node’s frequency and denoted as count. A mean HeteSim score for each source node was generated and used for further analysis of the source nodes. The top 10% of normalized and highly ranked source nodes were aggregated from the simulations (Figure 9). The top 10% of the source nodes were chosen based on the overall predicted relevance using the mean HeteSim score.

### 4.3. Analysis of Source Nodes Revealed by SemNet 2.0

#### 4.3.1. Functional Ontology Mapping

The biological process or function of the source nodes revealed by SemNet 2.0 simulations is not always available. Due to the vast simulation data, understanding the biological role of these source nodes through a literature survey may not be feasible. The SemNet 2.0 simulation generated a large amount of data. Thus, data-mining techniques were employed to map the source concepts to their biological functions. A common way of searching shared functions among genes is to incorporate the biological knowledge provided by biological ontologies [99,100,101]. The Gene Ontology resource is a major bioinformatics initiative that provides tools to annotate genes to their biological processes [102,103]. The mouse genome informatics (MGI) term mapper was used to provide ontologies or biological processes of the top 10% of genes or proteins [40,41]. Specifically, the list of identified source node names was input into the MGI Batch Query, and the respective functional ontology terms available were retrieved from the GO database. However, this method generated multiple functional classifications for a unique source node. Therefore, these functional ontologies were grouped by a unique ontology ID when duplicates or similar biological functions were listed. A unique numeric label was generated for each unique ontology ID (term label), and the frequency of each ontology term mapped to a source node was recorded (term count).

#### 4.3.2. Cytoscape

Cytoscape [38,39] is an open-source software platform for visualizing complex associations and integrating these with any attribute data. Cytoscape can build network models of interaction and tools for annotating and analyzing the connections or relationships in a data set [104]. The architecture is flexible, and the input data can include genes, proteins, chemicals, or enzymes [104]. Cytoscape [38,39] was used to generate a linked protein–protein network using the top 10% of identified source nodes and their mapped ontologies. The functional ontologies of each source node were analyzed. The functional ontologies that indicated a positive or negative relationship with a signaling molecule were considered. These relationships between source nodes and signaling molecules were selected by searching for specific keywords: “positive regulation” or “negative regulation”. A negative regulation in GO database terms refers to any process that stops, prevents, or reduces the frequency or rate of covalent alteration of one or more amino acid residues within a protein [103]. A positive regulation suggests any process that activates or increases the frequency or rate of chemical reactions and pathways involving a protein [103]. A protein–protein interaction (PPI) file was created to store these source nodes as inputs and signaling molecules as outputs using a simple interaction file (sif) format. The sif-formatted file consisted of three main column entries: (1) inputs, (2) interaction type, and (3) outputs. The interaction type used in Cytoscape was protein–protein interaction. The interaction edges between source nodes and signaling molecules were based on the specified positive or negative relationship using a +1 or −1 relation index, respectively. The PPI file was used to create the interaction network in Cytoscape. Two additional column entries were added to the PPI file—the edge relation indices and input node type—to visually distinguish between regulatory relationships, genes, and proteins. More information on generating the PPI file and the format can be found in the Cytoscape user manual [39].

## 5. Conclusions

This LBD study comprehensively and efficiently identified and prioritized relevant signaling molecules and pathways associated with DKD. Cross-domain relationships were queried and ranked from 33+ million PubMed articles using SemNet 2.0. The SemNet 2.0 analysis yielded two valuable outcomes: (1) the relatedness between source genes or proteins that intersect the DKD-GEC-IR pathophysiology; (2) the creation of a protein/gene interaction network using objective, comprehensive LBD findings in place of an inherently more limited manual literature review. EPHB4, SERPINB1, ITGB1 TYK2, CREG1, NFKBIA, SPI1, and SNAP23 were among the most highly ranked concepts at the intersection of the GEC, IR, and DKD domains. These findings corroborate the relevance of studying the synergistic interaction between the immune system and glomerular endothelial cells to better understand the early stages of DKD progression. The results support the use of LBD to aid in the prioritization of multi-scalar pathological mechanisms and drug targets, the development of protein–protein interactions and biochemical models, the testing of hypotheses through experiments, and the advancement of biomedical decision-making.

## Data Availability

The data and analysis files for this study are provided in a repository [82], which is available here: https://github.com/ashleefv/DKD_CaseStudy_SemNet2 (accessed on 13 March 2024). The code leverages the open-source software SemNet 2.0 [31,32], which is available in the following repository: https://github.com/pathology-dynamics/semnet-2 (accessed on 9 January 2024).

## Supplementary Materials

The following supporting information can be downloaded at: https://www.mdpi.com/article/10.3390/ijms25084503/s1.

## Appendix A

Table A1 provides a summary of previously published mathematical models to study inter-disease relatedness for DKD and the kidney more generally.

**Table A1:** Summary of mathematical models used to study inter-disease relatedness for DKD and the kidney more generally.

Topic	Data Type	Model Type	Main Contribution	References
DKD kidney and immune crosstalk	In vitro	Logic-based ODE model	Identified critical interactions and chemical species associated with the pathophysiology of glucose and immune-mediated glomerular endothelial dysfunction using a network-based model.	[9]
Glomerular fibrosis in DKD	Mice, in vitro	Kinetic ODE model	Clarified mechanisms behind diabetic glomerular fibrosis and the reasons behind the failure of certain drugs to guide the creation of better diabetes-related kidney damage therapies.	[15]
Hyperglycemia-induced podocyte injury in DKD	Human	Kinetic ODE model	Integrated mathematical modeling, optimization, and sensitivity analyses to simulate the effects of glucose on the local RAS in podocytes in diabetic nephropathy to achieve physiologically plausible model outputs.	[18]
Podocytes in kidney diseases	In vitro	Multi-compartment ODE model	Analyzed feedforward motifs, spatial dynamics, and the roles of the PKA and MAPK pathways and provided insights into factors that could drive proliferation or differentiation in cultured podocytes.	[19]
Tubulointerstitial inflammation and fibrosis in lupus nephritis	Human	PDE model	Modeled disease progression and effects of dosing levels for novel therapeutics by simulating various levels of inhibition necessary to attenuate the inflammation to the fibrosis pathway.	[20]
Kidney and cardiac disorders	Human	Kinetic ODE model	Used QSP approach to understand the complex interactions between cardiac and renal functions in HF-rEF and the effects of pharmacological interventions such as ACEi and SGLT2i therapies.	[22]
Kidney and bone metabolism	Human	QSP model	Network topology enabled more effective targeting of key components and better prediction of perturbation effects.	[24]
CKD and bone	Mixed	QSP model	Conjugated individual existing cardiac and renal models to study pathophysiological interplay and disease relatedness. This model can be used in personalizing therapy and research settings and generating new hypotheses.	[25]
Multiple myeloma and kidney disorders	Human	Kinetic ODE model	Captured the qualitative behavior of the cell and protein populations and their interaction with the proximal tubule of the kidney, free light chains, renal fibroblasts, and myeloma cells. This model may support better patient prognosis in patients with multiple myeloma and renal impairment.	[23]

ACE: angiotensin-converting enzyme. CKD: chronic kidney disease. DKD: diabetic kidney disease. HF-rEF: heart failure with reduced ejection fraction. i: inhibitor drug classes. ODE: ordinary differential equations. PDE: partial differential equations. QSP: quantitative systems pharmacology. RAS: renin–angiotensin system. SGLT: sodium–glucose co-transporters.

Figure A1, Figure A2, Figure A3 and Figure A4 are provided to show further analysis results.
